# Kinematic MRI Analysis of Reducible Atlantoaxial Dislocation for Decompression

**DOI:** 10.1155/2020/5395071

**Published:** 2020-12-15

**Authors:** Liang Dong, Chaoyuan Ge, Zhengwei Xu, Dongqi Wang, Honghui Sun, Dingjun Hao

**Affiliations:** The Department of Spine Surgery, Hong-Hui Hospital, Xi'an Jiaotong University College of Medicine, Xi'an 710054, China

## Abstract

**Background:**

Many doctors ignored the possibility that there is still a spinal cord compression (SCC) need for decompression after atlantoaxial reduction. Reduction can be achieved on kinematic magnetic resonance imaging (MRI); thus, we want to analyze the role of kinematic MRI in reducible atlantoaxial dislocation and make a preoperative decision whether to perform decompression.

**Methods:**

36 patients with atlantoaxial reduction on preoperative kinematic MRI in extension postures were enrolled retrospectively. Grouping was based on the condition of SCC after atlantoaxial reduction preoperatively. Group A: patients with SCC after atlantoaxial reduction on dynamic cervical MRI were treated with C1 laminectomy for decompression and atlantoaxial fixation. Group B: patients with no significant SCC, according to dynamic MRI, underwent only atlantoaxial fixation. Clinical outcomes were evaluated using JOA score for spinal cord function. Radiological outcomes were assessed by measuring spinal cord diameter on MRI.

**Results:**

The mean follow-up time was 17.1 months. Postoperative JOA score and percentage of SCC in both groups were significantly better than its preoperative score. There were no significant statistical differences in the JOA score at 12 months after surgery and the JOA improvement rate between two groups. All patients in the two groups had a lower percentage of SCC on preoperative extension MRI, compared with neutral MRI. No significant statistical differences in the spinal decompression improvement rate were observed between the two groups.

**Conclusions:**

Decompression should be performed in patients who still have significant SCC on preoperative kinematic MRI. Kinematic MRI could be used to assess SCC and decide whether to perform decompression preoperatively.

## 1. Introduction

Reducible atlantoaxial dislocation, as one of the types of atlantoaxial dislocation, has been described in many articles [1–3]. According to the above classification systems, atlantoaxial dislocation that achieves reduction in dynamic X-ray is classified as reducible atlantoaxial dislocation. Atlantoaxial reduction can be achieved by posterior atlantoaxial reduction and fixation. However, many doctors ignore the possibility of spinal cord compression (SCC) after complete atlantoaxial reduction.

Several articles have reported that there was still SCC caused by soft tissue mass, such as retroodontoid pseudotumor, which was frequently induced by inflammatory diseases as rheumatoid arthritis or repeated stress such as atlantoaxial dislocations [4, 5]. Although retroodontoid pseudotumor would be likely to cause SCC directly, some pseudotumors lead to slight or no compression after atlantoaxial reduction; decompression is not necessary in this situation. Nevertheless, it is difficult to assess that SCC is induced by retroodontoid pseudotumor or atlantoaxial dislocation on neutral MRI. Therefore, dynamic MRI can be performed to analyze the condition of SCC after atlantoaxial reduction. Our study is aimed at analyzing the role of dynamic MRI in determining whether to perform atlantoaxial decompression for reducible atlantoaxial dislocation.

## 2. Methods

### 2.1. Patient Population

In this retrospective study, 36 patients with atlantoaxial dislocation were enrolled between May 2013 and May 2019. Inclusion criteria were as follows: (1) patients with atlantoaxial dislocation and SCC appropriate for atlantoaxial fixation and fusion; (2) reducible atlantoaxial dislocation identified by dynamic X-rays. The etiology of atlantoaxial dislocation includes 12 cases of congenital odontoid nonunion, 5 cases of rheumatoid arthritis, and 19 cases of atlantoaxial fracture.

The operation of dynamic cervical MRI (Philips Achieva) in extension posture is as follows: the shoulder is raised with a high soft cushion to keep neck extension and to achieve atlantoaxial reduction. Dynamic MRI were performed for all patients. The following grouping was based on the condition of SCC after atlantoaxial reduction on dynamic MRI. The degree of SCC is indicated by the percentage of SCC (it is shown in the formula below). In group A, the percentage of SCC is greater than 20% after atlantoaxial reduction on dynamic MRI. All patients in group A underwent C1 laminectomy for decompression and atlantoaxial fixation and fusion. In group B, there was no significant SCC and the percentage of SCC is less than or equal to 20% after atlantoaxial reduction. All patients in group B underwent only atlantoaxial fixation and fusion. Symptoms and signs of the patients are shown in Table 1. The patient group included 4 men and 9 women (age range, 39–69 months; mean age, 52.1 months) in group A and 10 men and 13 women (age range, 41–74 months; mean age, 54.2 months) in group B (Table 1). However, grouping was not used for comparison; they, as two separate groups, are used to clarify the role of dynamic MRI in determining whether to perform decompression for different SCC.

### 2.2. Surgery Technique

All surgeries were performed by 2 senior surgeons (Hao DJ and Xu ZW) specialized in upper cervical surgery. The surgical decision was developed based on a syndromic, but not etiological, approach.

Neurological monitoring was performed, then general anesthesia was adopted, and the patient was kept in the prone position with the neck slightly flexed. The C1 posterior arch and C2 lamina were exposed. The C1 posterior arch entry point was approximately 20 mm lateral to the midline. According to preoperative 3D CT scans, the medial trajectory direction was approximately 10° and the cephalad direction was 5°. However, the optimal direction of the trajectory depends on the imaging orientation and intraoperative anatomy. C2 pedicle screws were also placed. If there was a high-riding vertebral artery, C2 pars screws were used. Two rods were then fixed to the screws rigidly, and atlantoaxial reduction was achieved on intraoperative fluoroscopy. Laminectomy of C1 was adopted for group A; autoiliac bone graft was modified to implant on the posterior rims of C1 and C2.

### 2.3. Outcome Evaluation

Postoperative CT scans and X-rays were used to assess the accuracy of screw placement and the condition of reduction. Complete reduction was defined as an atlantodental internal ≤ 3 mm. Japanese Orthopedic Association (JOA) scores and the JOA improvement rate were used to assess the improvement of spinal cord function.

(1) $$\begin{array}{c} \text{JOA improvement rate}=\frac{\text{postoperative JOA}–\text{preoperative JOA}}{17-\text{postoperative JOA}}\times 100\%\left(\text{postoperative JOA is defined as JOA score at }12\text{ months after surgery}\right). \end{array}$$

**Figure 1:**
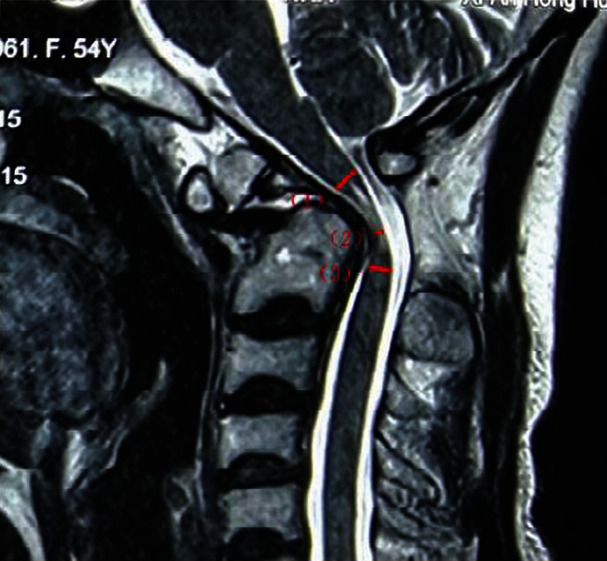
Sagittal neutral MRI showing an example of measurement of SCC: (1) normal spinal cord diameter above SCC; (2) narrowest spinal cord diameter; (3) normal spinal cord diameter below SCC. SCC: spinal cord compression.

Preoperative and postoperative spinal cord diameter on median sagittal neutral MRI was used to calculate the percentage of SCC and the improvement rate of SCC, which was used to evaluate the improvement rate of spinal cord diameter (Figure 1).

(2) $$\begin{array}{c} \text{Normal spinal cord diameter }\left(\text{mm}\right)=\frac{\text{normal spinal cord diameter above SCC}-\text{normal spinal cord diameter below SCC}}{2},\\ \text{Percentage of SCC}=\frac{\text{normal spinal cord diameter}-\text{narrowest spinal cord diameter}}{\text{normal spinal cord diameter}}\times 100\%. \end{array}$$

Percentage of SCC was also calculated in the condition of atlantoaxial reduction on dynamic MRI in extension postures.

(3) $$\begin{array}{c} \text{Improvement rate of spinal cord decompression}=\frac{\text{postoperative narrowest spinal cord diameter}-\text{preoperative narrowest cervical cord diameter}}{\text{normal spinal cord diameter}-\text{preoperative narrowest spinal cord diameter}}\times 100\%\left(\text{postoperative narrowest spinal cord diameter is the data at }12\text{ months after surgery}\right). \end{array}$$

**Figure 2:**
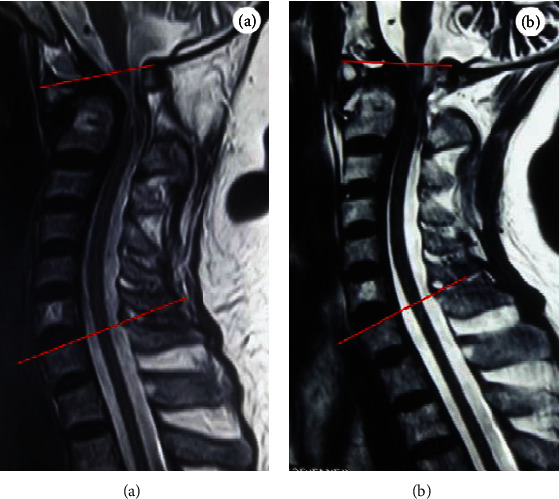
C1-7 Cobb angle is defined as the angle between the superior line of the C1 anterior arch and posterior arch and the C7 superior end plate: (a) the C1-7 Cobb angle on median sagittal neutral MRI; (b) the C1-7 Cobb angle on median sagittal extension MRI. The preoperative sagittal mobility of the cervical spine is defined as angle b − angle a.

The preoperative sagittal mobility of the cervical spine was defined as the C1-7 Cobb angle on median sagittal extension MRI with atlantoaxial reduction subtracted from the C1-7 Cobb angle on median sagittal neutral MRI (the C1-7 Cobb angle was the angle between the superior line of the C1 anterior arch and posterior arch and the C7 superior end plate) (Figure 2).

All the measurements were done independently at Honghui Hospital by three people (Dong L, Qian LX, and Chen XJ). The average value of measured data was imported into the above formulas.

### 2.4. Statistical Analysis

SPSS statistical software, version 19.0 (SPSS Inc., Chicago, IL) were performed. The paired *t* test was used to compare pre- and postoperative data. Two different groups were compared by the two-sample *t* test. *P* < 0.05 was considered statistically significant difference.

## 3. Results

All 36 patients were achieved atlantoaxial reduction on postoperative X-rays. There was no intraoperative spinal cord injury in all cases. According to the CT scans, 2 screws were inserted into the transverse foramen; fortunately, it did not induce vertebral artery injury. 3 patients had a high-riding vertebral artery on CT angiography imaging; six C2 pars screws were performed instead of C2 pedicle screws.

The mean follow-up was 16.6 ± 3.3 months (range, 12-22 months) in group A, and the mean follow-up was 17.5 ± 4.3 months (range, 12-28 months) in group B. At last follow-up, there was no screw loosening; all patients obtained atlantoaxial fusion except 1 patient, but this patient with atlantoaxial reduction and no screw loosening refused the revision surgery.

The following clinical outcomes are listed in Table 2. Postoperative JOA score in both groups was significantly better than preoperative JOA score (*P* < 0.01). Compared with the group A (before surgery, 5.5 ± 1.0; 1 month after surgery, 11.0 ± 1.4), the group B had significantly better JOA score preoperatively (7.6 ± 1.8) and at 1 month after surgery (12.0 ± 1.1) (*P* < 0.01). Nevertheless, there were no significant statistical differences in JOA score at 12 months after surgery (A: 13.2 ± 1.3% vs. B: 13.6 ± 1.2%; *P* = 0.32 > 0.05) and JOA improvement rate between the groups at 12 months after surgery (A: 66.0 ± 10.9% vs. B: 62.4 ± 15.2%, *P* = 0.34 > 0.05).

**Figure 3:**
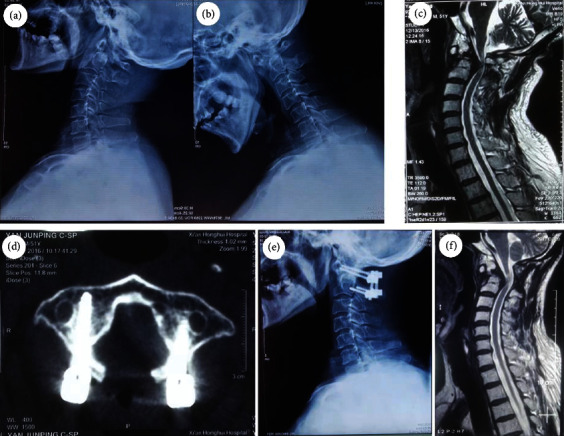
A 61-year-old female with single-level lumbar OVCF treated by unilateral PKP: (a, b) dynamic X-rays show atlantoaxial dislocation and reduction; (c) preoperative dynamic MRI shows evidence of spinal compression after atlantoaxial reduction; (d) postoperative CT shows atlas arch resection; (e) postoperative X-ray shows atlantoaxial reduction; (f) postoperative MRI shows spinal cord decompression.

**Figure 4:**
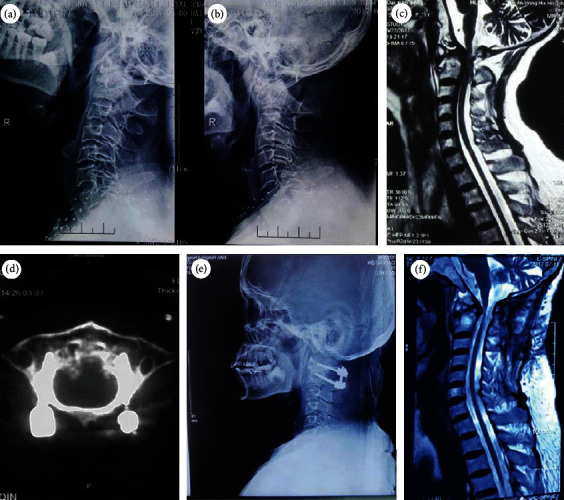
(a, b) Flexion-extension X-rays show atlantoaxial dislocation and reduction; (c) atlantoaxial reduction without spinal cord compression on preoperative dynamic MRI; (d) postoperative CT shows atlas arch conservation; (e) postoperative X-ray shows atlantoaxial reduction; (f) postoperative MRI shows spinal cord decompression.

The following radiological outcomes are listed in Table 2. All patients in the two groups had a lower percentage of SCC on extension MRI before surgery (*P* < 0.01), compared with that on neutral MRI. The percentage of SCC in group A significantly decreased from 63.6 ± 14.5% preoperatively to 9.1 ± 3.4% at 12 months after surgery (*P* < 0.01). Similarly, the percentage in group B also decreased from 51.8 ± 9.7% preoperatively to 5.1 ± 3.1% at 12 months after surgery (*P* < 0.01). No significant statistical differences in the spinal decompression improvement rate were observed between two groups at 12 months after surgery (A: 76.9 ± 9.6% vs. B: 74.8 ± 8.7%, *P* = 0.329 > 0.05). The preoperative sagittal mobility of the cervical spine in group A and group B was 19.2 ± 4.7 and 19.0 ± 6.1, respectively (*P* = 0.936 > 0.05) (Figures 3 and 4).

## 4. Discussion

The first atlantoaxial dislocation classification, which includes reducible and irreducible atlantoaxial dislocation, was reported by Greenberg [6]. Over the past several years, many different atlantoaxial dislocation classifications have been reported based on this classification [1–3]. According to these classifications, atlantoaxial dislocations that undergo reduction on dynamic X-rays are categorized as reducible atlantoaxial dislocation. In our study, the average sagittal mobility of the cervical spine between neutral MRI and extension MRI is 19.1°; all patients achieved atlantoaxial reduction on extension MRI. In the past several years, many different posterior screw fixation techniques for atlantoaxial dislocation have been introduced [7–9]. Reducible atlantoaxial dislocations are treated mainly by posterior atlantoaxial fixation according to the above classifications. All patients in our study achieved complete atlantoaxial reduction by using posterior atlantoaxial reduction and fixation.

However, almost all atlantoaxial dislocation classifications are based on the results of X-rays or CT scans, and some researchers investigated the bony vertebral canal on CT scans or X-rays to analyze the condition of SCC; effective spinal cord decompression seems to be achieved by increasing the width of the bony vertebral canal [10, 11]. Nevertheless, the bony vertebral canal is not available for some atlantoaxial dislocation with SCC caused by soft tissue mass, such as retroodontoid pseudotumors. Le Pape et al. showed that retroodontoid structures have been given different names, such as mass, cyst, pseudotumor, granuloma, and pannus [12]. Yu et al. [13] reported that rheumatoid arthritis plays an important role in the development of retroodontoid pseudotumors. Furthermore, repeated stress caused by excessive motion of the atlantoaxial joint plays a part in the development of this tumor, such as atlantoaxial dislocation [4]. In our study, the spinal bony canal was enlarged after atlantoaxial reduction; the percentage of SCC of all patients decreased on extension MRI, compared with neutral MRI. However, the group A patients still had a 38% percentage of SCC caused by retroodontoid pseudotumors after atlantoaxial reduction, and group B patients only had a 12% percentage of SCC.

In some articles, patients with retroodontoid pseudotumors underwent pseudotumor resection alone, but a new onset atlantoaxial dislocation and regrown pseudotumor led to SCC during the follow-up period [13]. Some investigators used the transoral or posterior approach to perform pseudotumor resection, which could have decompressed the spinal cord directly, but increased the risk of spinal cord injury and other complications [4, 14]. In recent years, atlantoaxial dislocation patients with retroodontoid pseudotumors were treated with C1–C2 fixation, in which the postoperative MRI showed reabsorption of the pseudotumor [4]. Consistent with the above articles, the percentage of SCC in group B decreased from preoperative 12% after atlantoaxial reduction to 5% at 12 months after surgery, which means that the small pseudotumor size was reduced after atlantoaxial fixation. Therefore, more than 20% percentage of SCC after atlantoaxial reduction was used as the grouping line to decompression in our study. The JOA score in group B also significantly increased at 12 months after surgery, compared with that before surgery and at 1 month after surgery.

Kakutani et al. suggested that C1 laminectomy was effective for spinal cord decompression and retroodontoid pseudotumor size reduction; C1 laminectomy also improves the blood flow of the pseudotumor and neurological function instantly [15]. In this study, we also performed C1 laminectomy for group A patients when the percentage of SCC is greater than 20% after atlantoaxial reduction. Our study also indicates that C1 laminectomy was effective for neurological improvement; the JOA score in group A increased instantly at 1 month after surgery; meanwhile, although there was still spinal cord compression in group A caused by the retroodontoid pseudotumor after surgery, which was embodied in the higher percentage of SCC at 12 months after surgery, compared with group B, the postoperative JOA score in group A had no statistical difference with that in group B at 12 months after surgery.

Limitations of this study were as follows: (1) more controlled studies should be performed to analyze whether only C1 laminectomy is sufficient for some patients with bigger soft tissue mass, then the mass causes more than 80% percentage of SCC; nevertheless, the maximum percentage of SCC after atlantoaxial reduction is 63.4% in our study; (2) we performed C1 laminectomy for retrospective cases with greater than 20% percentage of SCC; however, it is necessary to perform prospective controlled studies to assess whether only atlantoaxial fixation is also sufficient for some patients with greater than 20% percentage of SCC.

In conclusion, dynamic MRI can effectively analyze the condition of SCC after atlantoaxial reduction, especially for atlantoaxial dislocation patients with retroodontoid pseudotumors. Dynamic MRI can also be used to determine preoperatively whether to perform spinal cord decompression after atlantoaxial reduction. The atlantoaxial dislocation classification system should include dynamic MRI to establish a new subcategory of reducible atlantoaxial dislocation. Although our study concludes that C1 laminectomy improves spinal cord function, more controlled studies should be performed to compare the clinical efficacy of atlantoaxial fusion alone and atlantoaxial fusion with C1 laminectomy.

## Figures and Tables

**Table 1:** Patient clinical characteristics.

Clinical and radiological outcomes	*n* (total 36 patients)
*Symptoms and signs*	
Restricted neck movement	23/36
Neck pain	19/36
Distal upper-limb wasting	25/36
Quadriparesis	19/36
Ataxia	23/36
Dyspnea or sleep apnea	8/36
*Demographics*	
Group A (total 10 patients)	
Age (year)	52.1 (39-69)
Sex (men/women)	4/9
Follow-up time (month)	16.6 (12-22)
Group B (total 21 patients)	
Age (year)	54.2 (41-74)
Sex (men/women)	10/13
Follow-up time (month)	17.5 (12-28)

**Table 2:** Clinical evaluation parameters.

Parameters	Group A	Group B
*JOA*		
Preoperative	5.5 ± 1.0	7.6 ± 1.8^∗∗^
1 month	11.0 ± 1.4^a^	12.0 ± 1.1^∗∗^
12 months	13.2 ± 1.3^b^	13.6 ± 1.2
*JOA improvement rate (%)*	66.0 ± 10.9	62.4 ± 15.2
*Percentage of spinal cord compression (%)*		
Preoperative	63.6 ± 14.5	51.8 ± 9.7^∗∗^
Preoperative (atlantoaxial reduction)	38.6 ± 11.6^†^	12.1 ± 3.1^∗∗^
12 months	9.1 ± 3.4^††^	5.1 ± 3.1^∗∗^
*Spinal decompression improvement rate (%)*	76.9 ± 9.6	74.8 ± 8.7
*Preoperative cervical mobility (°)*	19.2 ± 4.7	19.0 ± 6.1

^a^Preoperative JOA vs. 1-month JOA, *P* < 0.05; ^b^12-month JOA vs. 1-month JOA, *P* < 0.05; ^∗∗^Group A vs. Group B, *P* < 0.05; ^†^Preoperative (atlantoaxial reduction) vs. preoperative, *P* < 0.05; ^††^12-month vs. preoperative (atlantoaxial reduction), *P* < 0.05.

## Data Availability

The data used to support the findings of this study are available from the corresponding author upon request.

## References

[1] Wang S., Wang C., Yan M., Zhou H., Dang G. (2013). Novel surgical classification and treatment strategy for atlantoaxial dislocations. *Spine*.

[2] Pruthi N., Nehete L. S. (2018). Use of intraoperative X-ray to differentiate between reducible versus irreducible atlantoaxial dislocation. *Surgical Neurology International*.

[3] Xu J., Yin Q., Xia H. (2013). New clinical classification system for atlantoaxial dislocation. *Orthopedics*.

[4] Barbagallo G. M., Certo F., Visocchi M., Palmucci S., Sciacca G., Albanese V. (2013). Disappearance of degenerative, non-inflammatory, retro-odontoid pseudotumor following posterior C1-C2 fixation: case series and review of the literature. *European Spine Journal*.

[5] Buttiens A., Vandevenne J., Van Cauter S. (2018). Retro-odontoid pseudotumor in a patient with atlanto-occipital assimilation. *J Belg Soc Radiol*.

[6] Greenberg A. D. (1968). Atlanto-axial dislocations. *Brain*.

[7] Harms J., Melcher R. P. (2001). Posterior C1-C2 fusion with polyaxial screw and rod fixation. *Spine*.

[8] Goel A., Laheri V. (1994). Plate and screw fixation for atlanto-axial subluxation. *Acta Neurochirurgica*.

[9] Tan M., Wang H., Wang Y. (2003). Morphometric evaluation of screw fixation in atlas via posterior arch and lateral mass. *Spine*.

[10] Nagaria J., Kelleher M. O., Mcevoy L., Edwards R., Kamel M. H., Bolger C. (2009). C1-C2 transarticular screw fixation for atlantoaxial instability due to rheumatoid Arthritis. *Spine*.

[11] Oshima S., Sudo H., Ito M., Abumi K. (2015). Subaxial sagittal alignment after atlantoaxial fixation techniques. *Journal of Spinal Disorders & Techniques*.

[12] Le Pape S., Gauthe R., Latrobe C., Leroux J., Roussignol X., Ould-Slimane M. (2016). Cervical myelopathy involving os odontoideum and retro-odontoid cyst treated with Harms C1-C2 arthrodesis. *Orthopaedics & Traumatology, Surgery & Research*.

[13] Yu S. H., Choi H. J., Cho W. H., Cha S. H., Han I. H. (2016). Retro-odontoid pseudotumor without atlantoaxial subluxation or rheumatic arthritis. *Korean Journal of Neurotrauma*.

[14] Tominaga H., Setoguchi T., Nagano S. (2016). Retro-odontoid mass without atlantoaxial instability causing cervical myelopathy: a case report of transdural surgical resection. *Spinal Cord Series And Cases*.

[15] Kakutani K., Doita M., Yoshikawa M. (2013). C1 laminectomy for retro-odontoid pseudotumor without atlantoaxial subluxation: review of seven consecutive cases. *European Spine Journal*.

